# The emergence of *Clostridium difficile* infection in Asia: A systematic review and meta-analysis of incidence and impact

**DOI:** 10.1371/journal.pone.0176797

**Published:** 2017-05-02

**Authors:** Nienke Z. Borren, Shadi Ghadermarzi, Susan Hutfless, Ashwin N. Ananthakrishnan

**Affiliations:** 1 Division of Gastroenterology, Massachusetts General Hospital, Boston, Massachusetts, United States of America; 2 University of Groningen, Groningen, The Netherlands; 3 Division of Gastroenterology & Hepatology, Johns Hopkins University, Baltimore, Maryland, United States of America; 4 Department of Epidemiology, Johns Hopkins Bloomberg School of Public Health, Baltimore, Maryland, United States of America; 5 Harvard Medical School, Boston, Massachusetts, United States of America; Cleveland Clinic, UNITED STATES

## Abstract

**Background:**

*Clostridium difficile* infection (CDI) is the most common healthcare associated infection and is highly prevalent in Europe and North America. Limited data is available on the prevalence of CDI in Asia. However, secular increases in prevalence of risk factors for CDI suggest that it may be emerging as a major cause of morbidity, highlighting the urgent need for a systematic study of the prevalence of CDI in Asia.

**Methods:**

We systematically searched PubMed/Medline and Embase for publications from Asia between 2000–16 examining prevalence of CDI. A random-effects meta-analysis was performed to calculate the pooled prevalence of *CDI* in Asia and to identify subgroups and regions at high risk.

**Results:**

Our meta-analysis included 51 studies from throughout Asia including 37,663 patients at risk among whom confirmed *CDI* was found in 4,343 patients. The pooled proportion of confirmed CDI among all patients with diarrhea was 14.8% with a higher prevalence in East Asia (19.5%), compared with South Asia (10.5%) or the Middle East (11.1%). There were an estimated 5.3 episodes of CDI per 10,000 patient days, similar to rates reported from Europe and North America. Infections due to hypervirulent strains were rare. CDI-related mortality was 8.9%.

**Conclusions:**

In a meta-analysis of 51 studies, we observed similar rates of *CDI* in Asia in comparison to Europe and North America. Increased awareness and improved surveillance of *Clostridium difficile* is essential to reduce incidence and morbidity.

## Introduction

*Clostridium difficile* infection (CDI) is the most common healthcare associated infection (HAI). Since its identification as the cause of pseudomembranous colitis in 1978[1], it has emerged as an important cause of morbidity particularly among hospitalized patients and led to epidemics with high mortality. An estimated 453,000 infections occur annually in the United States, 172,000 in Europe, and 18,005 in England.[2–4] Recognition of the burden of CDI has led to a multi-pronged strategy of provider education, institution of systematic testing, antibiotic stewardship and infection control programs which has blunted the rise, and even reduced the incidence of this infection in North America and Europe.[5, 6]

In contrast, little is known about the prevalence and impact of CDI in Asia as few systematic studies exist and testing remains infrequent, hampered by both a low index of clinical suspicion and the lack of readily available laboratory testing.[7] Yet, several factors favor the possible emergence of *C*. *difficile* as an important pathogen in Asia.[8, 9] While traditionally considered home to a young population, with improved life expectancy and control of other infectious diseases, many countries in Asia are witnessing an aging of their demographics and in most studies, the elderly are particularly susceptible to CDI.[10, 11] Chronic diseases, also a risk factor for CDI, have increased in prevalence, and so has the need for frequent healthcare contact and hospitalizations. In addition, antibiotic use, the strongest risk factor for CDI, is often indiscriminate and unregulated in some Asian countries.[12, 13]

Thus, there is an important need for systematic study of the prevalence and impact of CDI in Asia to inform both clinical practice as well as healthcare policy. We performed this systematic review and meta-analysis to (1) quantify the burden of CDI among countries in Asia; (2) identify subgroups and regions at high risk within this population; and (3) define the proportion of hypervirulent epidemic strains of *C*. *difficile*; and (4) quantify CDI-related mortality in comparisons to studies from the west.

## Methods

### Literature search

We conducted a systematic search of the MEDLINE/PubMed and EMBASE databases for studies providing prevalence or incidence rates of CDI in Asia. To quantify current burden, our search was limited to publications from January 2000 to June 2016. No language restrictions were applied in our search, but inclusion of the study in our full analysis required at least the abstract to be available in English. Our search strategy combined 3 different phrase groups by using the Boolean operator “AND” (S1 Table). The first search group consisted of terms relevant to identify CDI and included combination of “*Clostridium difficile*”, “*C difficile*” and “*C diff*” and *“Pseudomembranous colitis”*. The second group defining location included both broadly the phrase “Asia” as well as specific countries within Asia including China, Hong Kong, India, Iran, Israel, Japan, Korea, Malaysia, Singapore, Taiwan, Thailand and Turkey. The final search terms defined the outcome of interest and included “prevalence”, “incidence”, “epidemiology”, and “frequency”. The citation list from all eligible studies and reviews were also perused to identify other relevant studies.

### Inclusion and exclusion criteria

Studies were eligible for inclusion if they provided information on the incidence of *CDI* in Asia reported either as proportion of tests positive for toxigenic *C*. *difficile* among symptomatic patients testing, per 1000 hospital discharges, or per 10,000 patient days. Studies examining *C*. *difficile* carriage among asymptomatic individuals were excluded. Eligible studies could include either an inpatient or outpatient population.

### Data collection

The decision for inclusion of each study was made by two authors (NZB and ANA) who independently screened the studies by title and abstract. The following data were extracted from each study: year of publication, location, setting (inpatient or outpatient), population (nosocomial diarrhea, antibiotic-associated diarrhea, other), number of patients tested, and number of patients with confirmed CDI. The microbiological method for diagnosis of CDI was noted and where available, the results of molecular characterization for specific ribotypes. From each study, mean age prevalence of risk factors for CDI including exposure to antibiotics, use of proton pump inhibitors (PPI), and recent hospitalization were noted. Studies were grouped into three geographic regions: South Asia (India, Malaysia, Pakistan, Singapore, and Thailand), East Asia (China, Hong Kong, Japan, Korea, Taiwan) and the Middle East (Iran, Jordan, Kuwait, Lebanon, Qatar, Turkey). When necessary, attempts were made to contact the corresponding authors for additional pertinent information.

### Outcomes

The primary outcome of interest was expressed in one of three ways: (1) the proportion of tests positive for toxigenic *C*. *difficile* from among all patients with diarrhea; (2) rate of CDI per 1,000 admissions; and (3) the rate of CDI per 10,000 patient days. Our secondary outcome was CDI-associated mortality.

### Assessment of study quality

We used the Newcastle-Ottawa Quality Assessment Scale (NOS) to assess study quality. This scale ranks studies in 3 groups based on the selection of the cohort; the comparability of the cohorts; and the completeness of ascertainment of the outcome. Each study could receive up to 4 stars. Studies were considered representative if they consisted of an unselected group of patients and did not focus on individuals with specific comorbidities alone. Ascertainment bias was considered to be absent if all patients with diarrhea underwent similar testing strategies; reliance on clinical suspicion to trigger testing for select patients was deemed susceptible to bias.

### Statistical analysis

Heterogeneity between the studies was determined using the Cochran’s Q and I^2^ statistics. An I^2^ > 50% or p < 0.10 indicated significant heterogeneity. A DerSimonian and Laird random effects model was used for all analyses to determine the pooled prevalence rates (and 95% confidence intervals (CI)) for proportion of stool tests that were positive for *C*. *difficile* as well as rates per 1,000 admissions and 10,000 patient days. Pre-specified subgroup analysis was performed stratifying by setting, geographic region, and population under study (antibiotic-associated diarrhea, all nosocomial diarrhea). Publication bias was examined using the Egger test and visual examination of the funnel plot. Sources of heterogeneity between studies were identified by performing a meta-regression. All data were recorded in a Microsoft Excel spreadsheet (Microsoft Corp, Redmond, WA) and statistical analysis carried out using Stata 13.2 (StataCorp, College Station, TX).

## Results

### Literature search

**Fig 1:**
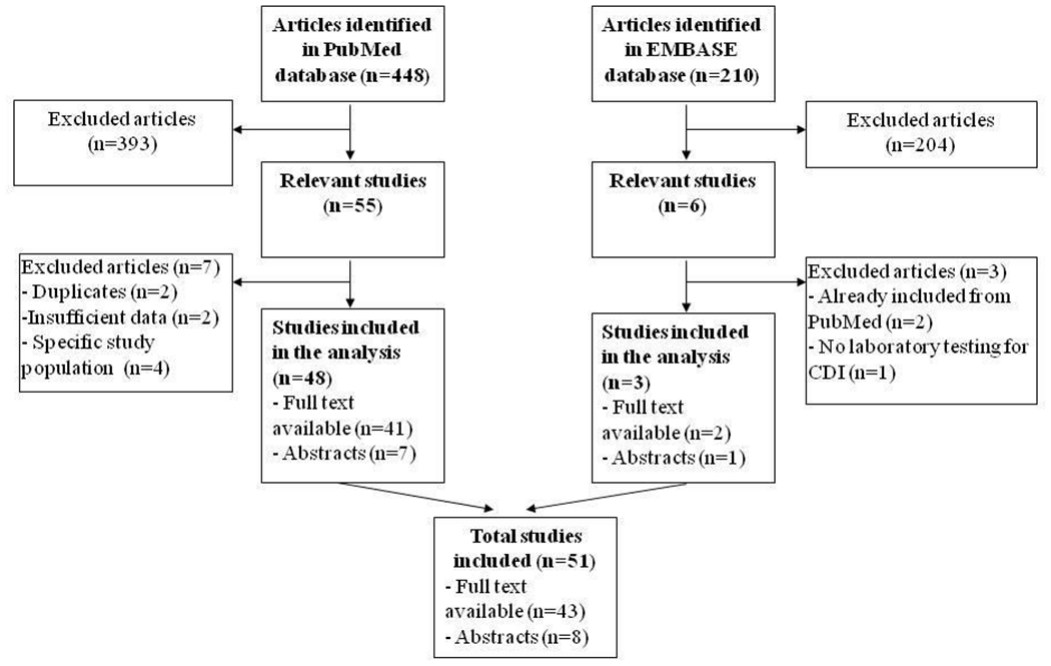
Flowchart of the literature search.

Our search of MEDLINE/Pubmed yielded 448 citations. Of these, 393 citations were excluded on initial screening of the title and abstract and the full text of remaining 55 articles were reviewed (Fig 1). Two articles representing duplicate data[14, 15], 2 with insufficient information[16, 17], and 4 examining *C*. *difficile* carriage in healthy individuals were excluded[18–21], resulting in a final cohort of 48 unique studies.[9, 22–71] Of these, only the abstract was available for review in English in 7 studies but sufficient relevant information could be extracted to allow for inclusion.[60–66, 71] A search on Embase yielded 3 additional studies that were eligible for inclusion. One large study from Thailand was not included as it did not include microbiological confirmation of CDI.[72]

### Study characteristics

The characteristics of the included studies are summarized in Table 1. Forty two included only hospitalized patients[9, 22–24, 28–40, 42, 43, 46, 48, 50–55, 57, 59–67, 69–71], 1 was exclusively among outpatients[47], and 8 included both groups.[25, 26, 41, 44, 45, 49, 56, 68] A total of 16 countries were represented, with China contributing the largest number of studies. Twenty-five studies were from East Asia[9, 22, 24, 26, 28, 34–37, 39–42, 44, 52–54, 56, 58–61, 67, 70, 71], 16 from South Asia[23, 25, 27, 29, 31, 33, 43, 46, 51, 55, 62–64, 66, 68, 69] and 10 from the Middle East.[30, 32, 38, 45, 47–50, 57, 65] The mean age of included patients was 60 years (data from 24 studies) and just fewer than half the cohort were women (43%, 34 studies). From 28 studies presenting data on antibiotic use; a mean of 84% of patients had been exposed recently (range 26–100%). Sixteen studies examining PPI use yielded a mean proportion of 49% (range 5–90%). From twenty-one studies where this data was available, the pooled proportion of recent hospitalization was 71% (range 19–100%).

**Table 1:** Characteristics of included studies.

Publication year	Author	Region	Setting	Study population	Study design	Diagnostic test(s) used	Number at risk	Number confirmed CDI	Proportion CDI +
2007	Kikkawa H	East Asia	Hospitalized patients	Patients with diarrhea	Prospective	Toxigenic culture and PCR	332	159	47.89
2008	Huang H	East Asia	Hospitalized patients	Patients with diarrhea	Prospective	Toxigenic culture, CCNA and PCR	587	56	9.54
2008	Shin BM	East Asia	Hospitalized patients	Patients with diarrhea	Retrospective	Toxigenic culture, EIA and PCR		285	27.00
2009	Cheng VC	East Asia	Hospitalized patients	Patients with diarrhea	Prospective	Toxigenic culture and PCR	496	37	7.46
2010	Chung CH	East Asia	Hospitalized patients	Patients with diarrhea	Retrospective	EIA	316	86	27.22
2010	Lee JH	East Asia	Hospitalized patients	Patients with diarrhea	Retrospective	Anaerobic culture and EIA		233	
2010	Lee YJ	East Asia	Hospitalized patients	Patients with diarrhea	Retrospective	Toxigenic culture and endoscopy		189	
2011	Cheng VC	East Asia	Unclear	Unclear	Retrospective	Toxigenic culture, CCNA and PCR	2440	307	12.58
2012	Lee YC	East Asia	Hospitalized patients	Patients with diarrhea	Prospective	Toxigenic culture, GDH and EIA	80	8	10.00
2012	Hung YP	East Asia	Hospitalized patients	Patients with diarrhea	Prospective	Toxigenic culture and PCR	168	7	4.17
2013	Kim J	East Asia	Hospitalized patients	Patients with AAD	Prospective	Toxigenic culture and PCR	769	166	21.59
2013	Hawkey PM	East Asia	Hospitalized patients	Patients with AAD	Retrospective	Toxigenic culture and PCR	70	21	30.00
2013	Kim YS	East Asia	Hospitalized patients	Patients with diarrhea	Retrospective	Toxigenic culture, EIA and endoscopy		1367	
2013	Han XH	East Asia	Hospitalized patients	Patients with diarrhea	Prospective	EIA	277	41	14.80
2014	Wang X	East Asia	Hospitalized patients	All patients	Prospective	Toxigenic culture and PCR	124	31	25.00
2014	Huang H	East Asia	Hospitalized patients	Patients with diarrhea	Prospective	Anaerobic culture, ccna, pcr	240	90	37.50
2014	Honda	East Asia	In- and outpatients	Patients with diarrhea	Retrospective	EIA	851	126	14.81
2014	Zhou FF	East Asia	Hospitalized patients	Patients with AAD	Prospective	Toxigenic culture, CCNA, PCR and endoscopy	206	63	30.58
2014	Fang WJ	East Asia	Hospitalized patients	Patients with diarrhea	Prospective	Toxigenic culture, EIA and PCR	400	82	20.50
2014	Ji D	East Asia	Hospitalized patients	All patients	Prospective	PCR	513	12	2.34
2014	Yang BK	East Asia	Hospitalized patients	Patients with diarrhea	Prospective	Toxigenic culture, PCR and endoscopy	1420	330	23.24
2014	Zhu Y	East Asia	Hospitalized patients	Patients with diarrhea	Prospective	Anaerobic culture and Toxigenic culture	277	41	14.80
2015	Choi HY	East Asia	In- and outpatients	Unclear	Retrospective	Unclear		2521	
2015	Galaydick J	East Asia	Hospitalized patients	Patients with diarrhea	Prospective	PCR	111	31	27.93
2016	Li Y	East Asia	Hospitalized patients	Patients with AAD	Prospective	EIA	470	93	19.79
2001	Shehabi AA	Middle East	Hospitalized patients	Patients with diarrhea	Prospective	EIA	300	29	9.70
2009	Ergen EK	Middle East	Hospitalized patients	Patients with diarrhea	Prospective	Toxigenic culture, PCR and Endoscopy	40	17	43.00
2010	Jamal W	Middle East	In- and outpatients	Patients with diarrhea	Prospective	Toxigenic culture, EIA and PCR	697	56	8.03
2010	Sadeqhifard N	Middle East	Hospitalized patients	Patients with diarrhea	Prospective	Toxigenic culture	942	57	6.05
2011	Nazemalhosseini-Mojarad E	Middle East	Outpatients	Patients with diarrhea	Prospective	EIA	356	19	5.34
2012	Khan FY	Middle East	In- and outpatients	Patients with diarrhea	Retrospective	EIA and Endoscopy		119	
2012	Jalali M	Middle East	In- and outpatients	Patients with diarrhea	Prospective	Anaerobic culture, Toxigenic culture and PCR	86	17	19.77
2014	Al-Thani AA	Middle East	In- and outpatients	Patients with diarrhea	Prospective	GDH, EIA and PCR	1532	122	7.96
2015	Moukhaiber R	Middle East	Hospitalized patients	Patients with AAD	Retrospective	Toxigenic culture, EIA and PCR	88	54	61.36
2015	Alinejad F	Middle East	Hospitalized patients	All patients	Prospective	EIA	37	8	21.62
2007	Koh TH	South Asia	Hospitalized patients	Patients with diarrhea	Prospective	Toxigenic culture, EIA and PCR	928	58	6.25
2008	Lim PL	South Asia	Hospitalized patients	Patients with diarrhea	Prospective	EIA	3508	386	11.00
2008	Chaudhry R	South Asia	Hospitalized patients	Patients with diarrhea	Retrospective	Toxigenic culture, EIA and PCR	524	37	7.06
2011	Hsu LY	South Asia	Unclear	Unclear	Prospective	EIA	7379	324	4.39
2011	Thipmontree W	South Asia	Hospitalized patients	Patients with diarrhea	Retrospective	Unclear	255	31	12.30
2011	Ingle M	South Asia	In- and outpatients	Patients with diarrhea	Retrospective	EIA	99	17	17.17
2012	Haider Naqvi SA	South Asia	Hospitalized patients	Patients with AAD	Retrospective	Toxigenic culture	191	57	29.84
2012	Kaneria MV	South Asia	Hospitalized patients	Patients with AAD	Cross-sectional	EIA	50	5	10.00
2012	Vaishnavi C	South Asia	Hospitalized patients	Patients with diarrhea	Prospective	Anaerobic culture and Toxigenic culture	79	5	6.33
2012	Hassan SA	South Asia	Hospitalized patients	Patients with diarrhea	Prospective	Toxigenic culture	175	24	13.71
2013	Ingle M	South Asia	In- and outpatients	Patients with diarrhea	Prospective	Toxigenic culture	150	12	8.00
2013	Vishwanath S	South Asia	Hospitalized patients	Patients with AAD	Prospective	Toxigenic culture and EIA	25	4	16.00
2015	Vaishnavi C	South Asia	Hospitalized patients	Patients with AAD	Prospective	Toxigenic culture and NAAT	1110	121	10.90
2015	Vaishnavi C	South Asia	Hospitalized patients	Patients with diarrhea	Retrospective	EIA	3044	533	17.51
2016	Thongkoom P	South Asia	Hospitalized patients	Patients with diarrhea	Retrospective	Unclear	5821	561	9.60
2016	Chau ML	South Asia	Hospitalized patients	Patients with diarrhea	Retrospective	GDH and NAAT	100	2	2.00

AAD—antibiotic associated diarrhea; CDI—Clostridium difficile infection, EIA—Enzyme immunoassay, GDH—glutamate dehydrogenase; NAAT—nucleic acid amplification testing; PCR—polymerase chain reaction

The included studies varied in the testing modality to determine *CDI*. The most commonly performed tests were anaerobic or toxigenic culture (71%) and the enzyme immunoassay (EIA) (52%). Nearly half the studies also reported using polymerase chain reaction / nucleic acid amplification test (PCR-NAAT) (51%). Other diagnostic tests used were cell culture cytotoxicity neutralization assay (CCNA) (8%), glutamate dehydrogenase assay (GDH) (6%), and lower gastrointestinal endoscopy (11%). Molecular characterization of *C*. *difficile* ribotype was performed in one-third of the included studies (n = 15). The mean proportion of infections due to ribotype 027 was 0.3% (range 0–2.1%) and from 12 studies, the proportion of ribotype 017 was 14% (range 0–48%).

### Proportion of C. difficile positivity

**Fig 2:**
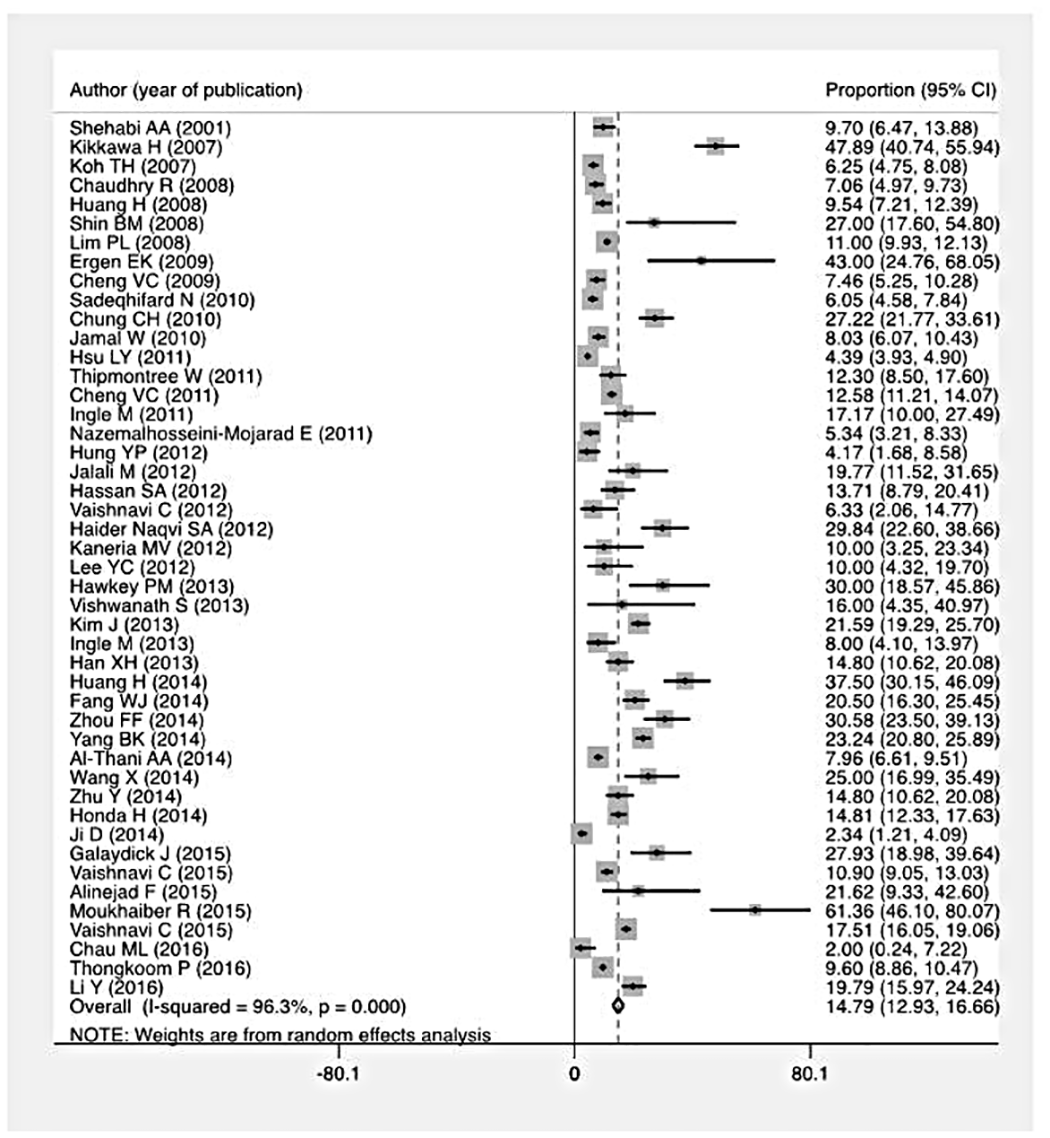
Forest plot of proportion of *C*. *difficile* positive tests among all patients tested.

This pooled analysis included 37,663 patients tested among whom CDI was confirmed in 4,343 patients. The overall pooled *C*. *difficile* positive rate was 14.8% (95% CI 12.9–16.7%) (Fig 2). However, there was significant heterogeneity between the studies (I^2^ = 96.3%, p<0.001), with rates from individual studies ranging from 2.0% to 61.4%. The pooled proportion of CDI was greater in the 37 studies restricted to hospitalized patients (16.4%, 95% CI 14.1–18.7%) than those with a mixed inpatient-outpatient population (11.1%, 95% CI 7.9–14.4%). The single study examining outpatients alone reported a significantly lower prevalence of 5.3% (p < 0.001)[47]. The prevalence of CDI was greater among studies restricting testing to antibiotic-associated diarrhea (25 studies, 20.9%) compared to all hospitalized patients with diarrhea (33 studies, 13.5%; p < 0.001).

**Fig 3:**
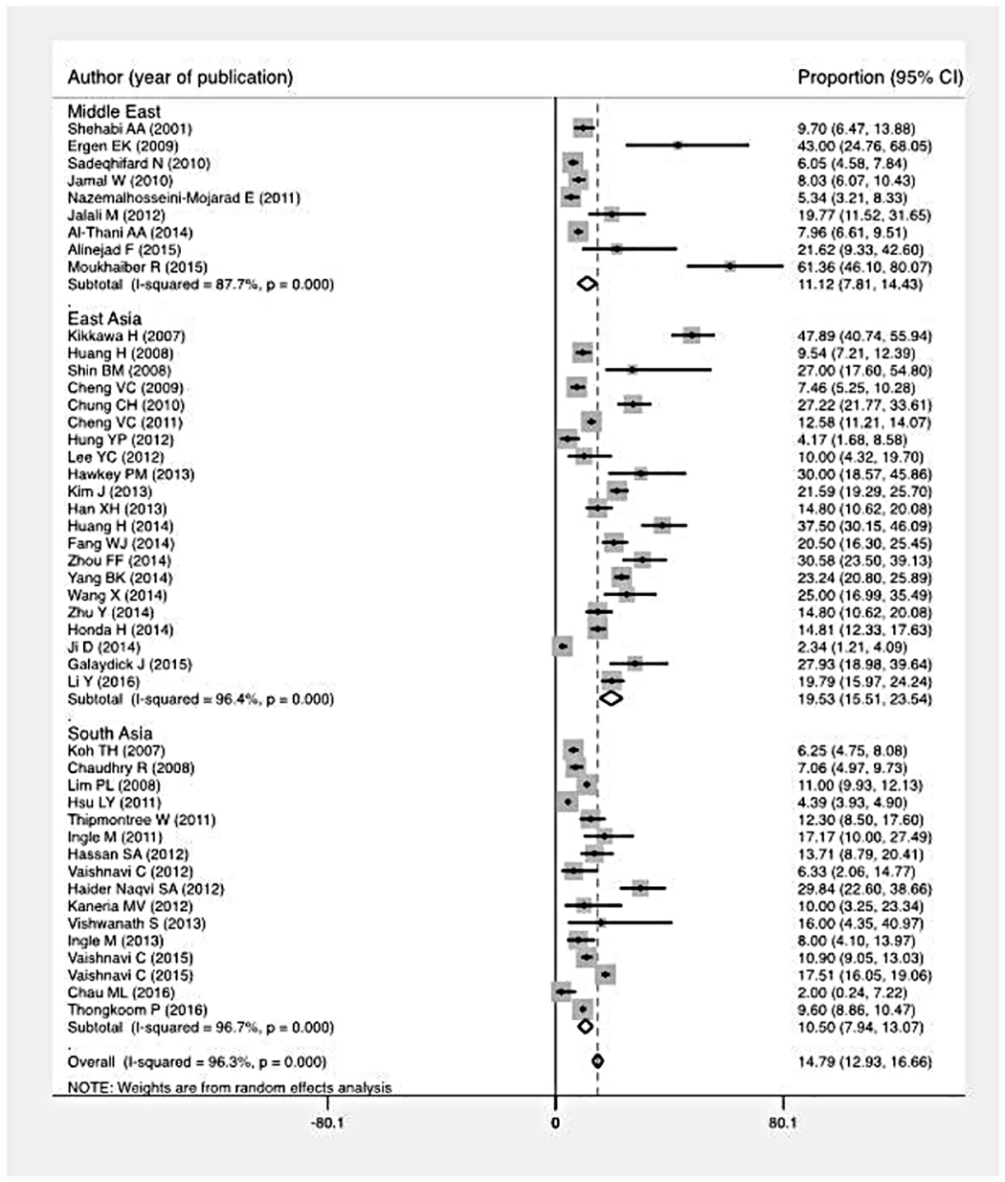
Forest plot of proportion of *C*. *difficile* positive tests among all patients tested, by region.

There was significant regional variation in occurrence of CDI. The proportion of *C*. *difficile* positivity was significantly higher among studies from East Asia (19.5%, 95% CI 15.5–23.5%, 21 studies) compared to those from the Middle East (11.1%, 95% CI 7.8–14.4%, 9 studies) or South Asia (10.5%, 95% CI 7.9–13.1%, 16 studies) (p < 0.001) (Fig 3, S1 Fig).

### Rates of C. difficile infection among hospitalized patients

**Fig 4:**
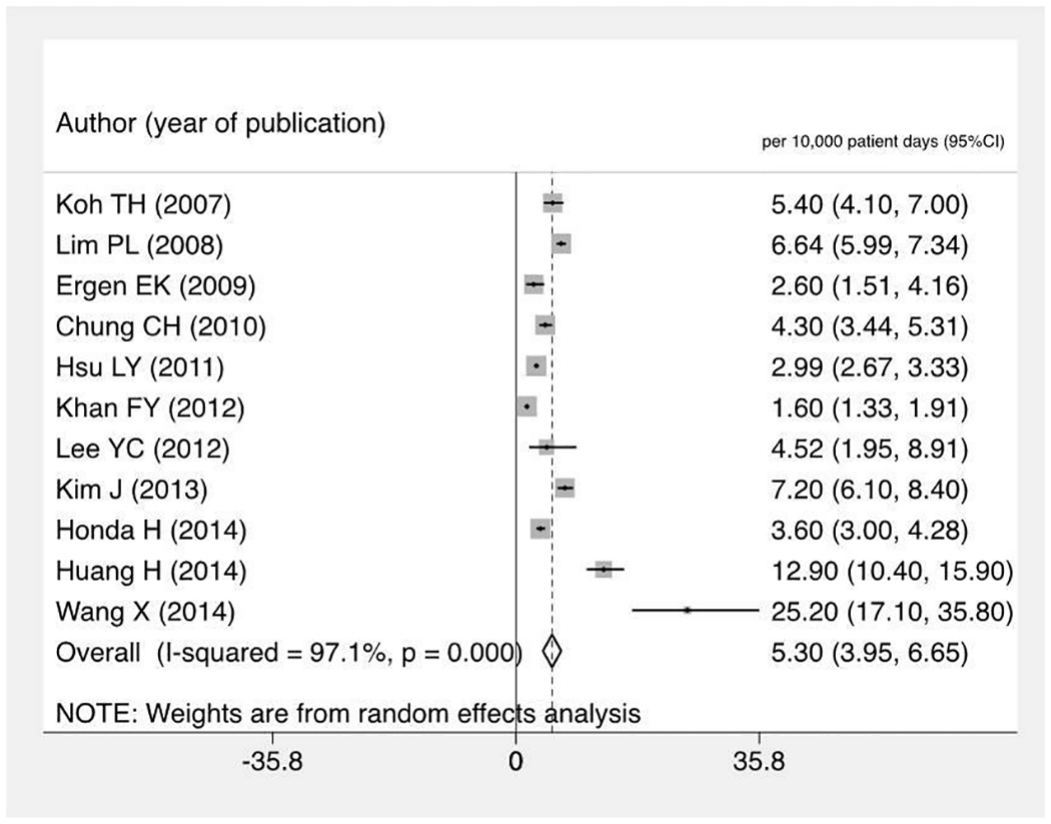
Forest plot of incidence rate of *C*. *difficile* infection per 10,000 patient days among hospitalized patients.

Eighteen studies provided extractable data on incidence per 1,000 admissions, yielding a pooled rate of 3.2 cases of CDI per 1,000 admissions (95% CI 2.4–3.9). However there was significant heterogeneity between the studies (I^2^ = 99.3%). Eleven studies provided sufficient information to estimate the incidence rate of CDI per 10,000 patient days. This yielded a pooled incidence of CDI of 5.3 per 10,000 patient-days (95% CI 4.0–6.7) (Fig 4). Excluding one study conducted exclusively in a high risk ICU population did not significantly alter the pooled incidence (4.9 per 10,000 patient days).[36]

### Outcomes of CDI

Thirteen studies provided information on CDI-related mortality (range 30–180 days).^22,24,28,36,41,44,45,49,57–60,62^ The random effects pooled rate of CDI-related death was 8.9% (95% CI 5.4%– 12.3%).

### Meta-regression

As most studies reported proportion of *C*. *difficile* positive tests as their outcome, meta-regression to identify influential covariates were performed for this outcome. Only geographic region of origin achieved statistical significance while there was a trend towards significance for the proportion of patients recently (S2 Table). Study setting, number of included patients, year of study, and proportion of patients exposed to antibiotics or PPI were not associated with rate of CDI. Specifically, we also did not identify a temporal trend over time in the proportion of stool tests that were positive for *C*. *difficile* (S2 Fig).

### Study quality and publication bias

S3 Table presents the quality scores for the included studies. While not all fields of the NOS were applicable for our meta-analysis, all studies were deemed of adequate quality to be included in the analysis. Begg and Egger tests both showed significant likelihood of publication bias (p = 0.046 and p<0.001, respectively) (S3 Fig).

## Discussion

The contribution of CDI to morbidity and mortality among hospitalized patients is well recognized in North America and Europe. However, little is known about whether *C*. *difficile* is equally prevalent and consequential in Asia. A systematic study of this is important to not only accurately quantify the burden of CDI in a population witnessing an increase in many of its risk factors, but also essential to inform disease surveillance and interventions to prevent the dramatic rise in incidence noted elsewhere.

Our systematic review demonstrated a pooled prevalence of CDI of 14.8% among all patients tested and 16.4% among hospitalized patients with diarrhea. These findings are similar to the estimates from other regions. In a multi-country European surveillance study, the proportion of stool samples positive for *C*. *difficile* ranged from 4 to 39%[73]. A multicenter study by the Centers for Disease Control (CDC) in the United States performing surveillance for *C*. *difficile* revealed a similar rate of positive tests, ranging from 7% to 20%.[74] In a nationwide study from Spain analyzing 807 stool specimens, 7.8% were found to be positive for *C*. *difficile*.[75]

The pooled incidence rate of CDI from Asia in our meta-analysis was 5.3 per 10,000 patient days. This is similar to reports from western countries. In a multicenter study from Europe, the incidence of CDI was reported as 4.1 per 10,000 patient days.[73] In the EUCLID study, the mean incidence rate was similarly 7 per 10,000 patient days with estimates from individual countries varying from 0.7 to 28.7.[76] A nationwide systematic study from Spain placed the rate at 3.8 per 10,000 patient-days.[75] The incidence is similar in the United States with a median hospital-onset CDI rate of 5.4 per 10,000 patient days.[77] As well, the pooled CDI-related mortality rate of 8.9% is also comparable to western estimates; for example, Lessa *et al*. reported a mortality rate of 6.4% in a systematic study from the United States.[78] Thus, despite the perception of CDI being uncommon in Asia, our findings suggest that the incidence and impact is similar to that noted in the West.

Among studies were molecular characterization of CDI was performed, the prevalence of hypervirulent ribotype 027 was only 0.3%. In comparison, this ribotype accounted for 21% of all *C*. *difficile* isolates[79] in the CDRN and 19% in the European EUCLID study.[76] While literature is not uniformly consistent on the impact of this strain, it has been associated with higher levels of toxin production and hypervirulence, [80, 81] leading to outbreaks initially in Canada and subsequently.[81] While the low prevalence among isolates in Asia is reassuring, the high rates of fluoroquinolone use in this population and the resistance of the ribotype 027 strain to this antibiotic class makes it essential to conduct regular surveillance for this strain.[13, 82]

There are several implications to our findings. Despite the recognition of CDI as an important HAI, there remains the perception of it being infrequent or inconsequential in Asia. In contrast to this, our results demonstrate both an incidence and mortality comparable to the west. Two narrative reviews, by Collins *et al*.[8] and Burke et al.[83] similarly emphasized the lack of awareness of CDI among physicians, that, along with our findings here, highlight the urgent need for education of healthcare professionals in Asia about its burden and impact. There is the need for appropriate infection control methods within hospitals including hand washing, contact isolation, minimization of unnecessary and over the counter dispensation of antibiotics, and development of antibiotic stewardship programs to reduce risk of CDI and prevent emergence of epidemic strains. The need for such measures attains additional urgency as several epidemiologic trends including aging of the population and growing burden of chronic disease favor escalation of CDI in Asia.

We readily acknowledge several limitations to evidence base contributing to our study. First, there was significant heterogeneity between the studies. However, a similar wide variation in incidence has also been observed across hospitals and between countries in the West. The heterogeneity was not completely explained by region, period of study, sample size, or testing strategy suggesting that either true variability in prevalence of CDI or the effect of other unmeasured factors. Second, nearly all the studies were conducted in a hospitalized setting and most did not differentiate community acquired from hospital acquired infection. As community acquired CDI may contribute to up to one-third of all CDI[84, 85], there is a need to systematically examine its occurrence globally. Only a few studies performed molecular characterization; there is the need for more robust data to accurately define the prevailing strains in Asia. Fourth, we observed evidence of publication bias and few studies reported on the impact of CDI on mortality or need for surgery. Finally, we searched the two most widely used medical literature databases—Embase and Pubmed—for relevant studies. However, we acknowledge that studies published solely in a regional language in Asia, particularly in smaller non-indexed journals, may not be comprehensively captured by these databases.

In conclusion, in this systematic review, we document that the burden of *CDI* in Asia is similar to that identified in North America and Europe. This highlights the need not only for further examination of the impact of *C*. *difficile* in this understudied geographic region but also the urgent need to educate providers about its consequence. There is also an important need for institution of appropriate measures to reduce the risk for development and transmission of this infection to reduce its adverse impact on patient outcomes.

## Supporting information

S1 Table Electronic search strategy for Pubmed. (DOC)

S2 Table Meta-regression analysis to identify covariates influencing proportion of *C*. *difficile* positivity. AAD—antibiotic associated diarrhea; EIA—Enzyme immuno assay; PCR—polymerase chain reaction.
(DOC)

S3 Table Quality of included studies using the Newcastle-Ottawa Quality Assessment Scale. (DOC)

S1 Checklist PRISMA checklist for meta-analyses. (DOC)

S1 Dataset Minimally identified dataset used for analysis. (XLS)

S1 Fig Geographic distribution of proportion of tests positive for *Clostridium difficile* within Asia. (JPG)

S2 Fig Secular trend in the proportion of stool tests that were positive for *C*. *difficile*. (JPG)

S3 Fig Funnel plot for assessment of publication bias. (JPG)
