# Supplementary material for: The emergence of Clostridium difficile infection in Asia: A systematic review and meta-analysis of incidence and impact
Source: PLoS One. 2017 May 2;12(5):e0176797. doi: 10.1371/journal.pone.0176797 (PMC5413003; doi:10.1371/journal.pone.0176797)
Supplement: S1 Table — (DOC) [file pone.0176797.s001.doc]

**S1 Table: Electronic search strategy for Pubmed**

#1: ("clostridium difficile"[MeSH Terms] OR ("clostridium"[All Fields] AND "difficile"[All Fields]) OR "clostridium difficile"[All Fields]) OR (C[All Fields] AND difficile[All Fields]) OR (C[All Fields] AND diff[All Fields]) OR ("enterocolitis, pseudomembranous"[MeSH Terms] OR ("enterocolitis"[All Fields] AND "pseudomembranous"[All Fields]) OR "pseudomembranous enterocolitis"[All Fields] OR ("pseudomembranous"[All Fields] AND "colitis"[All Fields]) OR "pseudomembranous colitis"[All Fields])

#2: ("asia"[MeSH Terms] OR "asia"[All Fields]) OR ("china"[MeSH Terms] OR "china"[All Fields]) OR ("hong kong"[MeSH Terms] OR ("hong"[All Fields] AND "kong"[All Fields]) OR "hong kong"[All Fields]) OR ("india"[MeSH Terms] OR "india"[All Fields]) OR ("iran"[MeSH Terms] OR "iran"[All Fields]) OR ("israel"[MeSH Terms] OR "israel"[All Fields]) OR ("japan"[MeSH Terms] OR "japan"[All Fields]) OR ("korea"[MeSH Terms] OR "korea"[All Fields]) OR ("malaysia"[MeSH Terms] OR "malaysia"[All Fields]) OR ("singapore"[MeSH Terms] OR "singapore"[All Fields]) OR ("taiwan"[MeSH Terms] OR "taiwan"[All Fields]) OR ("thailand"[MeSH Terms] OR "thailand"[All Fields]) OR ("turkey"[MeSH Terms] OR "turkey"[All Fields])

#3: ("epidemiology"[Subheading] OR "epidemiology"[All Fields] OR "prevalence"[All Fields] OR "prevalence"[MeSH Terms]) OR ("epidemiology"[Subheading] OR "epidemiology"[All Fields] OR "incidence"[All Fields] OR "incidence"[MeSH Terms]) OR ("epidemiology"[Subheading] OR "epidemiology"[All Fields] OR "epidemiology"[MeSH Terms]) OR ("epidemiology"[Subheading] OR "epidemiology"[All Fields] OR "frequency"[All Fields] OR "epidemiology"[MeSH Terms] OR "frequency"[All Fields])

**Final search**: #1 AND #2 AND #3
