# Supplementary material for: The emergence of Clostridium difficile infection in Asia: A systematic review and meta-analysis of incidence and impact
Source: PLoS One. 2017 May 2;12(5):e0176797. doi: 10.1371/journal.pone.0176797 (PMC5413003; doi:10.1371/journal.pone.0176797)
Supplement: S2 Table — AAD—antibiotic associated diarrhea; EIA—Enzyme immuno assay; PCR—polymerase chain reaction. (DOC) [file pone.0176797.s002.doc]

**S2 Table: Meta-regression analysis to identify covariates influencing proportion of *C. difficile* positivity**

| **Variable** | **Studies, n** |  | **Co-efficient (95%CI)** | **P value** |
| --- | --- | --- | --- | --- |
| Region (ref = South Asia) | 46 | East Asia | 8.4 (1.5 to 15.4) | 0.017 |
|  |  | Middle East | 5.5 (-3.4 to 14.5) | 0.225 |
| Setting (ref = hospitalized patients) | 44 | Outpatients | -12.1 (-33.8 to 9.6) | 0.275 |
|  |  | In- and outpatients | -4.9 (-14.7 to 4.6) | 0.304 |
| Study population (ref = all diarrhea) | 43 | Patients with AAD | 6.6 (-1.4 to 14.6) | 0.108 |
|  |  | All patients | 0.5 (-12.0 to 13.0) | 0.935 |
| Year of study start | 42 |  | 0.3 (-0.7 to 1.4) | 0.551 |
| Exposure to antibiotics | 25 |  | -0.01 (-0.17 to 0.15) | 0.889 |
| Exposure to proton pump inhibitor | 15 |  | 0.06 (-0.12 to 0.25) | 0.532 |
| Recently hospitalized | 20 |  | -0.16 (-0.32 to -0.0004) | 0.050 |
| Diagnostic test (ref= EIA) | 43 | PCR | -5.81 (-20.4 – 80.79) | 0.424 |
|  |  | Culture | 3.77 (-5.51 – 13.06) | 0.416 |

AAD – antibiotic associated diarrhea; EIA – Enzyme immuno assay; PCR – polymerase chain reaction
