# Supplementary material for: The emergence of Clostridium difficile infection in Asia: A systematic review and meta-analysis of incidence and impact
Source: PLoS One. 2017 May 2;12(5):e0176797. doi: 10.1371/journal.pone.0176797 (PMC5413003; doi:10.1371/journal.pone.0176797)
Supplement: S3 Table — (DOC) [file pone.0176797.s003.doc]

**S3 Table: Quality of included studies using the Newcastle-Ottawa Quality Assessment Scale**

| **Year of Publication** | **Author** | **Representativeness** | **Ascertainment** | **Not present at start of study** | **Assessment** | **Score** |
| --- | --- | --- | --- | --- | --- | --- |
| 2001 | Shehabi AA | 1 | 0 | 1 | 1 | 3 |
| 2007 | Kikkawa H | 1 | 0 | 1 | 1 | 3 |
| 2007 | Koh TH | 1 | 0 | 1 | 1 | 3 |
| 2008 | Huang H | 1 | 1 | 1 | 1 | 4 |
| 2008 | Lim PL | 1 | 0 | 1 | 1 | 3 |
| 2008 | Chaudhry R | 1 | 1 | 1 | 1 | 4 |
| 2008 | Shin BM | 1 | 1 | 1 | 1 | 4 |
| 2009 | Cheng VC | 1 | 0 | 1 | 1 | 3 |
| 2009 | Ergen EK | 1 | 1 | 1 | 1 | 4 |
| 2010 | Chung CH | 1 | 1 | 1 | 1 | 4 |
| 2010 | Lee JH | 1 | 0 | 1 | 1 | 3 |
| 2010 | Lee YJ | 1 | 0 | 1 | 1 | 3 |
| 2010 | Jamal W | 1 | 1 | 1 | 1 | 4 |
| 2010 | Sadeqhifard N | 0 | 1 | 1 | 1 | 3 |
| 2011 | Cheng VC | 1 | 0 | 1 | 1 | 3 |
| 2011 | Nazemalhosseini-Mojarad E | 1 | 0 | 1 | 1 | 3 |
| 2011 | Hsu LY | 0 | 0 | 1 | 1 | 2 |
| 2011 | Thipmontree W | 1 | 1 | 1 | 1 | 4 |
| 2011 | Ingle M | 1 | 1 | 1 | 1 | 4 |
| 2012 | Lee YC | 0 | 1 | 1 | 1 | 3 |
| 2012 | Hung YP | 1 | 1 | 1 | 1 | 4 |
| 2012 | Khan FY | 1 | 1 | 1 | 1 | 4 |
| 2012 | Jalali M | 1 | 1 | 1 | 1 | 4 |
| 2012 | Haider Naqvi SA | 0 | 0 | 1 | 1 | 2 |
| 2012 | Kaneria MV | 0 | 1 | 1 | 1 | 3 |
| 2012 | Vaishnavi C | 1 | 0 | 1 | 1 | 3 |
| 2012 | Hassan SA | 0 | 1 | 1 | 1 | 3 |
| 2013 | Kim J | 1 | 1 | 1 | 1 | 4 |
| 2013 | Hawkey PM | 0 | 0 | 1 | 1 | 2 |
| 2013 | Kim YS | 1 | 1 | 1 | 1 | 4 |
| 2013 | Han XH | 0 | 1 | 1 | 1 | 3 |
| 2013 | Ingle M | 1 | 1 | 1 | 1 | 4 |
| 2013 | Vishwanath S | 1 | 1 | 1 | 1 | 4 |
| 2014 | Wang X | 0 | 1 | 1 | 1 | 3 |
| 2014 | Huang H | 1 | 1 | 1 | 1 | 4 |
| 2014 | Honda H | 1 | 1 | 1 | 1 | 4 |
| 2014 | Zhou FF | 0 | 1 | 1 | 1 | 4 |
| 2014 | Fang WJ | 0 | 0 | 1 | 1 | 2 |
| 2014 | Ji D | 0 | 0 | 1 | 1 | 2 |
| 2014 | Al-Thani AA | 1 | 1 | 1 | 1 | 4 |
| 2014 | Yang BK | 1 | 1 | 1 | 1 | 4 |
| 2014 | Zhu Y | 0 | 0 | 1 | 1 | 2 |
| 2015 | Choi HY | 1 | 0 | 1 | 1 | 3 |
| 2015 | Galaydick J | 1 | 1 | 1 | 1 | 4 |
| 2015 | Moukhaiber R | 0 | 0 | 1 | 1 | 2 |
| 2015 | Alinejad F | 0 | 0 | 1 | 1 | 2 |
| 2015 | Vaishnavi C | 1 | 1 | 1 | 1 | 4 |
| 2015 | Vaishnavi C | 1 | 0 | 1 | 1 | 3 |
| 2016 | Li Y | 0 | 1 | 1 | 1 | 3 |
| 2016 | Thongkoom P | 1 | 0 | 1 | 1 | 3 |
| 2016 | Chau ML | 0 | 0 | 1 | 1 | 2 |

Higher score indicates higher study quality
