# Supplementary figures and images for: The emergence of Clostridium difficile infection in Asia: A systematic review and meta-analysis of incidence and impact
Source: PLoS One. 2017 May 2;12(5):e0176797. doi: 10.1371/journal.pone.0176797 (PMC5413003; doi:10.1371/journal.pone.0176797)

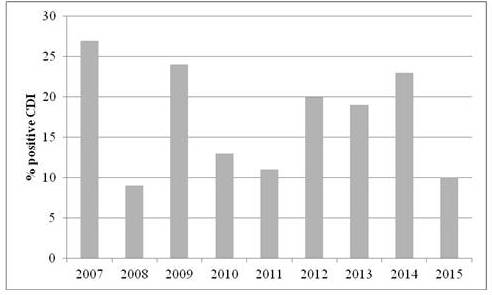

Supplement: S1 Fig — (JPG) [file pone.0176797.s006.jpg]

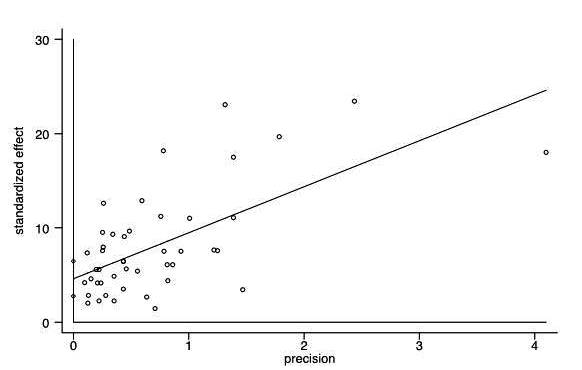

Supplement: S2 Fig — (JPG) [file pone.0176797.s007.jpg]

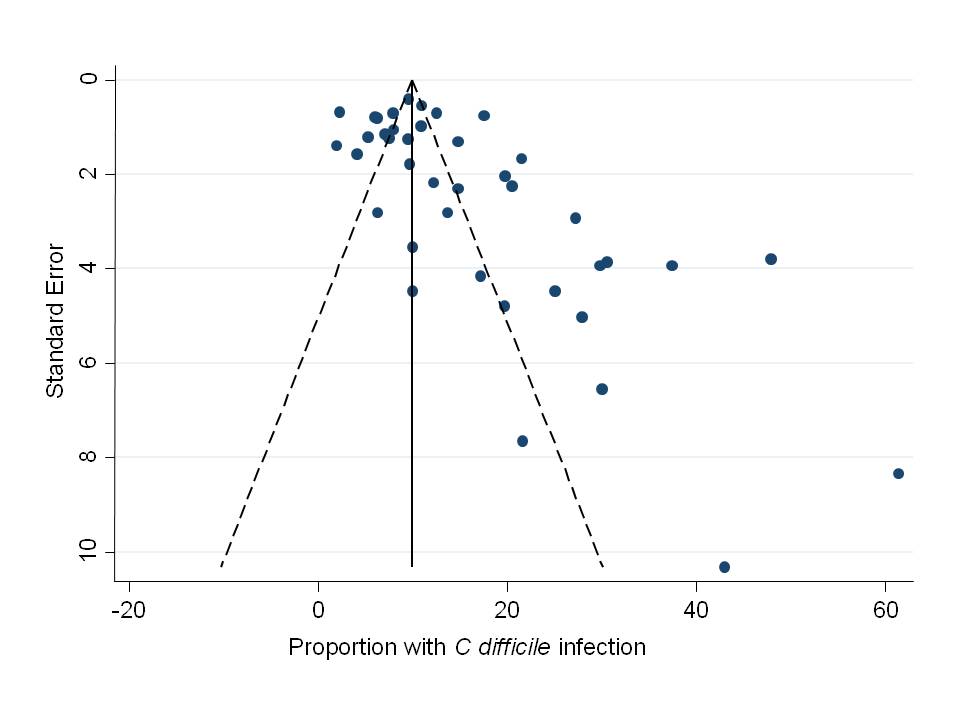

Supplement: S3 Fig — (JPG) [file pone.0176797.s008.jpg]
